# Positive psychology and employee adaptive performance: systematic literature review

**DOI:** 10.3389/fpsyg.2024.1417260

**Published:** 2024-08-14

**Authors:** Guihong Tang, Raida Abu Bakar, Safiah Omar

**Affiliations:** Department of Management and Marketing, Faculty of Business and Economics, University of Malaya, Kuala Lumpur, Malaysia

**Keywords:** positive psychology, psychological capital, PERMA, engagement, self-efficacy, adaptive performance

## Abstract

Adaptive performance will increasingly be confronted with new insights as society today changes constantly. This raises questions as to what factors will impact employee’s adaptive performance and what is their inner psychological mechanism. The terms of positive psychology and adaptive performance are important concepts in the domain of organizational behavior and human resource development areas. The literature, however, lacks a systematic review of it. Our research seeks to explore the inherence of employee adaptive performance via the prism of positive psychology, including Psychological Capital and PERMA (Positive Emotions, Engagement, Relationships, Meaning and Accomplishment). We selected 27 papers out of 382, which were generated from Web of Science and Scopus databases associated the keywords of the two concepts, and used the 2020 PRISMA flow program for the paper screening. By analyzing the underpin theories, the causation, and the measurement, we discovered that there is a complex and nuanced relationship between positive psychology and adaptive performance, and most of the research to date suggests that positive psychology components improve employee adaptive performance. This study maps the current knowledge at the nexus of positive psychology and adaptive performance to identify existing gaps and potential for further investigation.

## 1 Introduction

The current state of the global and technical environment has become more complex, confusing, and dynamic. Working in an era of complex demand that professionals need to be prepared to use their extensive experience bases, develop new knowledge (Mylopoulos et al., 2018) and quickly acquire new skills when required. In most areas, people will have to keep coming up with new ideas and changing the way things are seen, and people who can handle these changes are known as “adaptive experts” in the literature (van Tartwijk et al., 2023). For example, in the food and beverage industry, it is imperative for enterprises and their employees to swiftly respond to the dynamic nature of customer demands and preferences in order to optimize customer satisfaction and gain a competitive edge (Reig-Botella et al., 2024). Additionally, since the COVID-19 pandemic crisis has made the workplace more uncertain and unpredictable, it is essential that we examine at potential approaches to enhance employee’s motivation and adaptive performance (Junça-Silva and Menino, 2022).

Adaptive performance-“employees’ ability to adapt to fast-changing work conditions” (Ilgen and Pulakos, 1999), therefore has gain a better comprehension of the capabilities and performance of employees in the face of ever-evolving circumstances (Jundt et al., 2015; Park and Park, 2019). This line of research is anticipated to offer guidance to employers on how to foster employee expertise and capacity development that is most appropriate for the new work environments (Jundt et al., 2015).

There are many factors that influence adaptive performance. In their review article, Park and Park (2019) break down these factors into four categories: individual, job, group and organization. However, as talent becomes increasingly crucial in today’s business environment, the importance of individual is getting more significant, as innovation, productivity and customer satisfaction are all dependent on talent (Sondhi and Nirmal, 2013). In numerous disciplines, the effect of experts’ motivation is becoming more widely acknowledged (Wang and Wang, 2021), which is crucial and cost-effective. Employee’s motivation is significantly determined by their psychology states (Chintalapti, 2021), therefore, to improve the positive psychology of the employees is essential in nowadays workplace.

Nearly a decade ago, in a paper titled “The Future of Positive Psychology,” Seligman and Csikszentmihalyi (2000) called for “positive psychology” should include a study of human well-being, happiness, excellence and optimal human functioning. There’s no denying the importance of positive psychology in improving organizational performance and it’s become increasingly popular in recent years (Seligman et al., 2005). Positive psychology emphasizes human qualities, such as positive attributes and individual strengths, which has been widely accepted as having a beneficial effect in fostering an organizational culture that appreciates the potential of individuals (Peterson and Spiker, 2005). In recent years, positive psychology has seen a surge in popularity and has been utilized at a variety of levels, however, its application to the workplace and its impact to talent’s work performance has not been as widely explored. It would be highly intriguing to investigate the potential and the numerous advantages that positive psychology can bring to adaptive performance. Additionally, there is a lack of consensus regarding the methodology used, the theoretical frameworks adopted, and the location and identity of the investigation’s topics (Vada and Prentice, 2022). Therefore, this research is trying to conduct a systematic literature review (SLR) of research on adaptive performance from the prism of positive psychology and to provide the literature gaps for future studies.

We extract and analyze their findings across relevant results from existing papers, this is a straightforward and well-organized process to search for and locate several peer-reviewed publications on connected research issues in the same field of study (Kraus et al., 2022). An overview is employed to lay out the evidence that is currently available and pinpoint literature gaps (Lunny et al., 2018). The research on employee adaptive performance in the area of positive psychology is summarized in our overview of reviews.

The research questions are:

RQ1. What are the theories underlying positive psychology’s application to adaptive performance?
RQ2. What is the causality between positive psychology and adaptive performance?
RQ3. What positive psychology factors and adaptive performance measurements are used in this type of research?

In order to evaluate employee adaptive performance research within the context of positive psychology, we structure our study in a systematic manner. RQ1 is essential for comprehending the underlying presumptions utilized to create the adaptive performance conceptualization as it sheds light on the theoretical underpinnings of positive psychology and the methods employed by researchers to establish these linkages. RQ2 is important because determining the cause-effect relationship is an essential part of method development for employee adaptive performance research. RQ3 is a critical factor in determining the optimal selection of positive psychology constructs for adaptive performance. This research utilizes a systematic literature review methodology to address these questions. The remaining portions of this study are organized as follows: The research methods used in the literature review are described in Section 2, the bibliographic commentary of the prior literature is presented in Section 3, the content of the literature is analyzed in Section 4, the study is concluded with a summary of the findings in Section 5, and the limitations, identified gaps in the literature, and areas for future research are listed in Section 6.

## 2 Research method

### 2.1 Inclusion and exclusion criteria of the study

By employing a systematic literature review, this research builds on the work of Tranfield et al. (2003) and Xiao and Watson (2019). “Adaptive performance” here refers the capacity to adapt and modify one’s behavior as a result of changes in circumstances or information. It is characterized by adaptability, the ability to learn from experiences, and the capacity to adjust to new situations (Zheng et al., 2020; Liu et al., 2021). We consider “positive psychology” to encompass all facets of one’s inner resources, such as virtues, psychic powers, self-discipline, resilience, self-efficacy, optimism, hope and self-confidence (Chintalapti, 2021). Numerous contexts, such as psycho-oncology, education, and the workplace, can benefit from the use of positive psychology (Galanakis and Tsitouri, 2022). Observing the research objectives, the inclusion criteria of this study should be papers that investigate the relation between positive psychology and “adaptive performance” in workplace, including keywords of “psychological capital” (Optimism”, “Resilience”, “Hope”, “Self-efficacy”) and “PERMA” (“positive emotion”, “engagement”, “relationship”, “meaning”, “accomplishment”) and their dimensions. While duplication, papers not written in English, conference reviews, papers with unrelated substance, and so forth are examples of exclusion criteria.

### 2.2 Search strategy

This research uses the Web of Science and Scopus databases to compile articles on positive psychology and adaptive performance. These two databases are the most reputable platforms for this study since they allow researchers to map excellent research papers from many fields. In addition to the user-friendly nature of the databases, it is possible to access a comprehensive set of research article profiles through the use of discipline-specific keywords and search terms (Tranfield et al., 2003). The first keywords enter (“positive psychology” OR “PERMA” OR “positive emotion” OR “engagement” OR “relationship” OR “meaning” OR “accomplishment” OR “psychological capital” OR “PsyCap” OR “Optimism” OR “Resilience” OR “Hope” OR “Self-efficacy”) AND (“work adaptive performance” OR “employee adaptive performance” OR “adaptive performance”) includes in titles and abstracts all the terms linked to positive psychology and adaptive performance. The papers are collected on 6 July 2024. To find the most relevant publications, we do not employ additional search parameters such as the publication date. Furthermore, according to the 2020 version of PRISMA framework (Haddaway et al., 2022), we use the same keywords to search pertinent publications in our prior research and records.

### 2.3 Study selection process

With the strain set, we obtain 185 papers from Web of Science, 197 papers from Scopus, and a selection process starts with a total of 382 publications. 131 papers are found duplicated in step 1 and after removing, there are 251 left. Step 2 reports are screened via title and abstract. The publications that most closely match the research goals and have a high probability of contributing to the RQs are found in this stage. The terms “adaptive performance” and “positive psychology,” as well as their synonyms, bring up 47 papers after our initial search. Upon the closer examination, total 204 documents have been determined that in question are related to no abstract can be retrieved, conference papers and conference reviews, topics on physics and engineering, medical research, migrants’ adaptation, leadership, training or even sports; none of which apply to this study as they are not towards the individual adaptive performance in the workplace. Table 1 is the summary of these 204 publications that are categorized as inappropriate:

**Table 1:** Category of inappropriate papers.

Category	Number of Papers
Abstract cannot be obtained	2
Aeronautics and astronautics	4
Book chapter	8
Conference paper/Conference review	12
Education	12
Leadership	30
Medical research	24
Other management areas (Big 5, cognition, job crafting, organization, etc.)	55
Others: biology/telecommunication/AI/Environment	9
Physics and engineering	16
Research on social phenomena	16
Sports	3
Training and Learning	13
**Total**	**204**

After the abstract filtering, we select 47 most relevant articles to this study. The results of the search were then filtered based on the criteria of inclusion and exclusion. We restrict our search to complete English-language articles written in the workplace. This implies excluding studies which without full texts, do not write in English, not in workplace, and are not published in good journal, means that the journal which cannot be found by the SCImago Journal and Country Rank. The relationships between positive psychology and adaptive performance, including their dimensions, are explained in the paper’s content and papers answering the research questions are considered to meet the inclusion criterion. Following this last filter, we obtain the 27 articles that are most pertinent, which are published between 1989 and 2024 (Table 2).

**Table 2:** Inclusion and Exclusion process for the full-text paper selection.

Inclusion Criteria (keywords)	Number	Exclusion Criteria	Number
Positive emotion, adaptive performance	1	Duplication paper (sources: WOS, Scopus, previous record, citation)	131
Engagement, adaptive performance	10	Abstract cannot be retrieved	2
Meaning, adaptive performance	3	Not related by screened with the title and abstract	202
Psychological capital, adaptive performance	2	Paper full text cannot be retrieved	2
Resilience, adaptive performance	3	Paper does not be written in English	3
Optimism, adaptive performance	1	Papers which published in journals that cannot be found by SJR	3
Self-efficacy, adaptive performance	7	Paper content is not related to the topic and research questions	9
		Systematic literature review papers	3
**Total**	**27**		**355**

**Figure 1:**
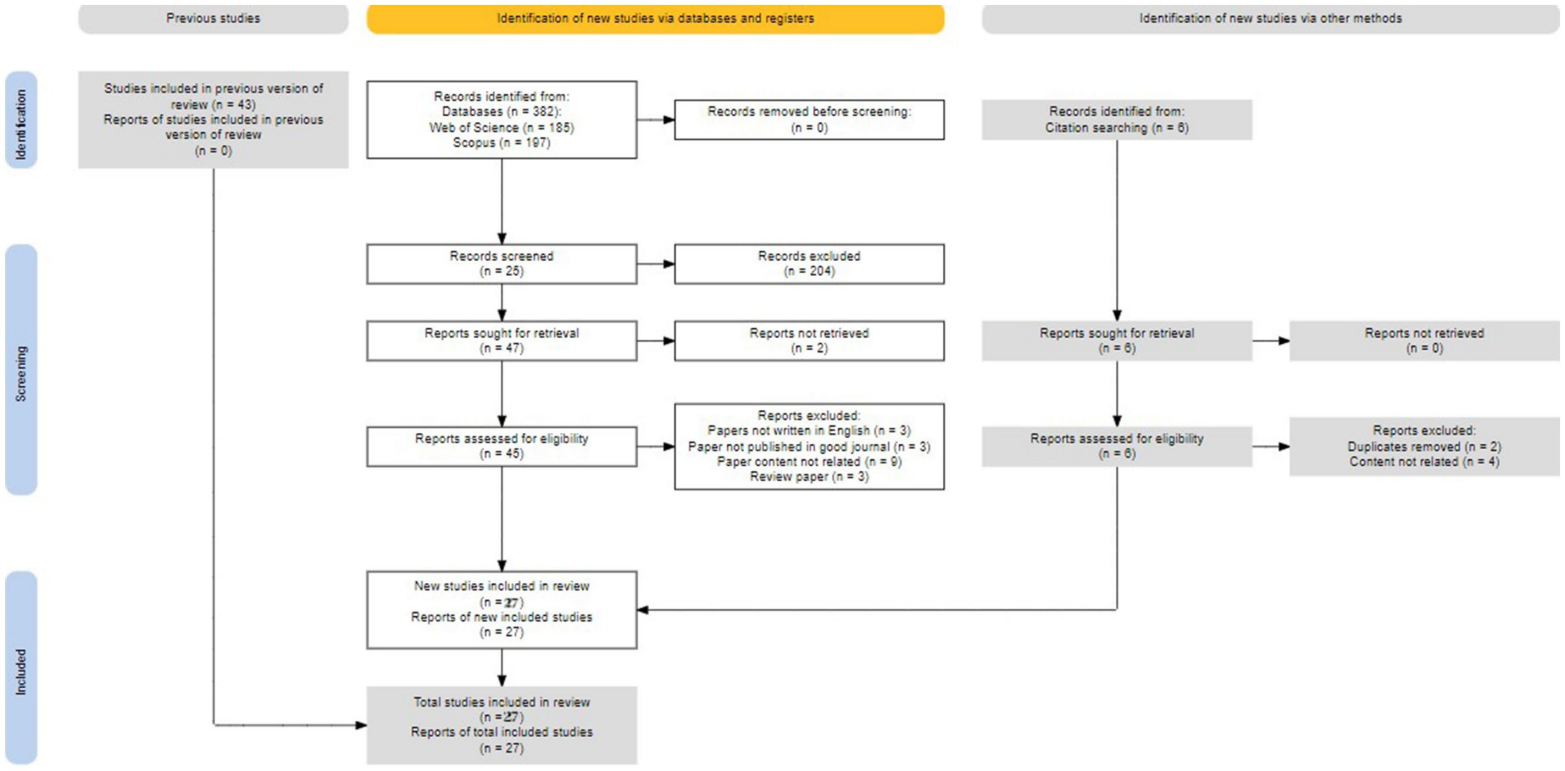
Publication PRISMA flow diagram (Haddaway et al., 2022).

According to the requirement of 2020 version of PRISMA, records identified from database is 382, prior studies and records have been screened and checked as well, 43 papers are considered related after keywords searching, but none of them apply to this research after full content examination. 6 papers from citation appear to relate to the topic but 0 of them has been selected due to duplication or not as relevant as expected. Table 2 demonstrates the detail of the whole inclusion and exclusion process, and Figure 1 indicates the decision-making process of paper selection in PRISMA.

## 3 Bibliographic analysis

### 3.1 The positive psychology and adaptive performance publication trends

**Figure 2:**
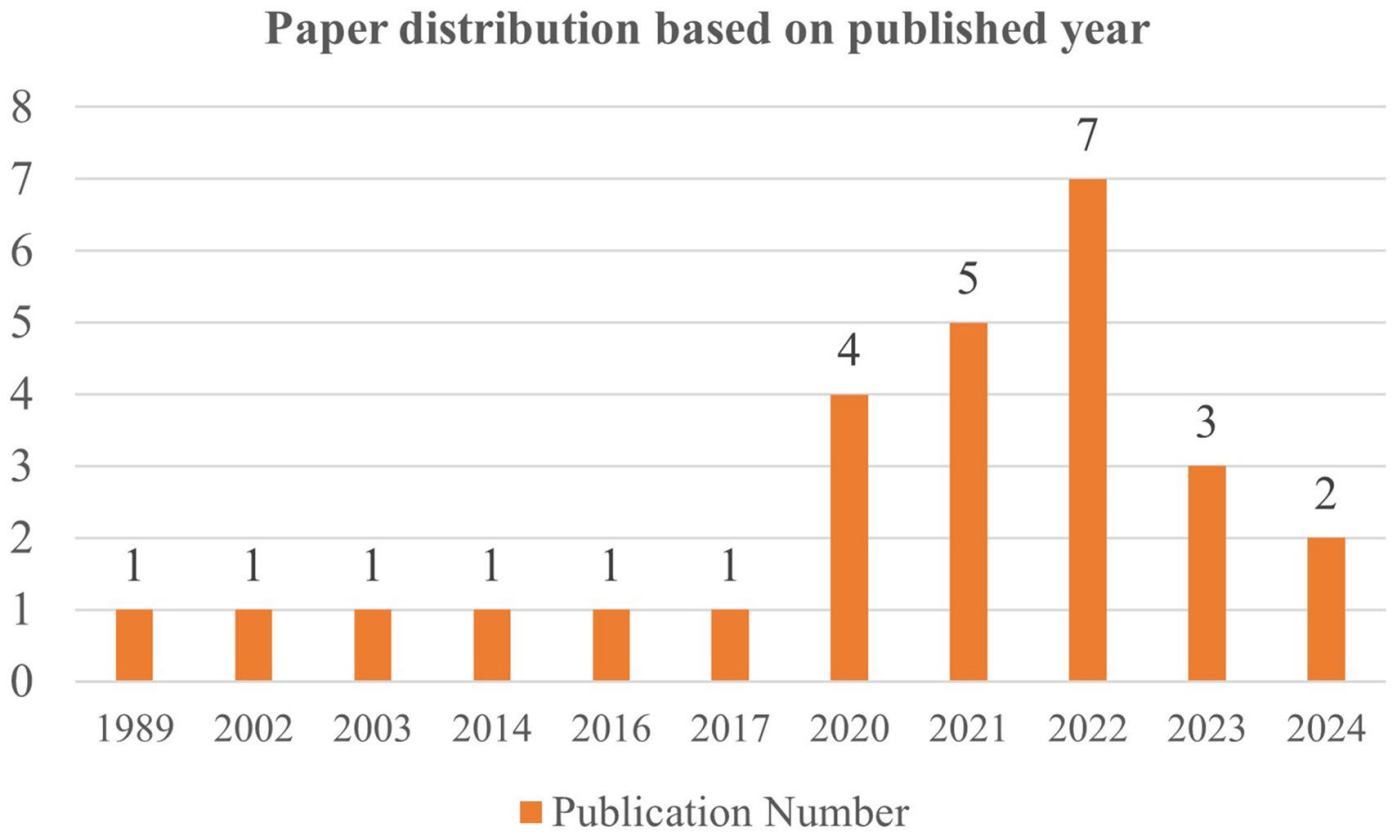
Distribution of papers based on publication year.

We discover that the earlier research done on positive psychology and adaptive performance conducted in the China (11.11%, *n* = 3), India (11.11%, *n* = 3), Malaysia (7.41%, *n* = 2), United States (7.41%, *n* = 2), Turkey (7.41%, *n* = 2), Korea (7.41%, *n* = 2), the rest (48.15%, *n* = 13) are from Finland, France, Dubai, Indonesia, The Netherlands, Pakistan, Saudi Arabia, United Kingdom, Spain and South Africa, etc. It reveals that this topic has attracted attention around the world although the publication number is still few. With the majority of studies appearing between 2020 and 2022, the focus on positive psychology and adaptive performance has been continuously increasing since 1989 (Figure 2).

Our article results are based on the previously mentioned 6 July 2024, Web of Science and Scopus search date. Consequently, we evaluate the frequency of the publications provided in this work with caution. Based on the most recent ranking supplied by SJR (SCImago Journal and Country Rank) Best Quartile Year 2023, we summarize the journals in Table 3. According to the SJR Best Quartile report, majority of the articles we review (51.85%, *n* = 14) are published in journals with a WOS-Q2 index, while the remaining papers are published in journals with a WOS-Q1 index (25.93% *n* = 7), Scopus-Q4 index (11.11%, *n* = 3) and 1 paper each with WOS-Q3, WOS-Q4 and Scopus Q3.

The 27 peer-reviewed studies (Table 4) are published in 22 different journals. The majority of the articles have been published in the top management journals, including Human Performance, Journal of Hospitality and Tourism Management and Journal of Managerial Psychology. As we anticipate, research with a global setting is common. It indicates that change is becoming more widespread around the world and to motivate employees to adapt to the challenge is getting more and more essential (Yang et al., 2022; Akyürek et al., 2023). It often involves the use of engagement, self-efficacy, meaning and other factors to achieve the objective and leading to the adaptive performance result of the professionals, bring cost-efficacy to the organizations (Kossek and Perrigino, 2016), and eventfully boosting the development of the companies (Reig-Botella et al., 2024).

**Table 3:** Percentage of journal sources.

Category	Quantity	Percentage
WOS Q1	7	25.93%
WOS Q2	14	51.85%
WOS Q3	1	3.70%
WOS Q4	1	3.70%
Scopus-Q3	1	3.70%
Scopus-Q4	3	11.11%
**Total**	27	100.00%

**Table 4:** Existing articles and their ranking.

Item	Journal	QTY	Source	SJR Rank 2023	Publish Year
1	Administrative Sciences	2	WOS	Q2	2021 & 2024
2	Australian Journal of Psychology	1	WOS	Q1	2003
3	Behavioral Sciences	1	WOS	Q2	2023
4	BRQ-business Research Quarterly	1	WOS	Q1	2022
5	Economies	1	WOS	Q2	2022
6	European Journal of Tourism Hospitality and Recreation	1	WOS	Q4	2023
7	Human Performance	1	WOS	Q1	2002
8	Human Resource Development Quarterly	1	WOS	Q2	2021
9	International Area Studies Review	1	WOS	Q3	2014
10	International Journal of Management	1	Scopus	Q4	2020
11	International Journal of Innovation Management	1	WOS	Q2	2020
12	International Journal of Productivity and Performance Management	1	WOS	Q2	2023
13	International Journal of Supply and Operations Management	1	Scopus	Q3	2021
14	Journal of Applied Behavioral Science	1	WOS	Q2	2021
15	Journal of Hospitality and Tourism Management	1	WOS	Q1	2022
16	Journal of Managerial Psychology	1	WOS	Q1	2016
17	Journal of Work and Organizational Psychology	1	WOS	Q2	2024
18	Personality and Individual Differences	1	WOS	Q1	2022
19	Psychologist-manager Journal	1	Scopus	Q4	2017
20	Scandinavian Journal of Behaviour Therapy	1	Scopus	Q4	1989
21	Spanish Journal of Psychology	1	WOS	Q1	2020
22	Sustainability	5	WOS	Q2	2020 &2021 &2022*3

### 3.2 Research design distribution

The primary objective of the majority of these papers is to investigate and evaluate adaptive performance via positive psychology. Consequently, it is to be expected that quantitative research techniques will predominate in the literature. 92.59% of the 27 studies examine adaptive performance by employing the quantitative approach (*n* = 25), which include a variety of technical techniques like explanatory and models that are predictive. The related research is largely based on the use of archive methods, with a few experiments. Another paper (3.70%) use qualitative methods, the remaining 1 paper (3.70%) uses mixed method for analysis. These papers focus on the mechanism of positive factors and how they affect employee behavior and their adaptive performance.

### 3.3 Distribution based on dimensions

**Figure 3:**
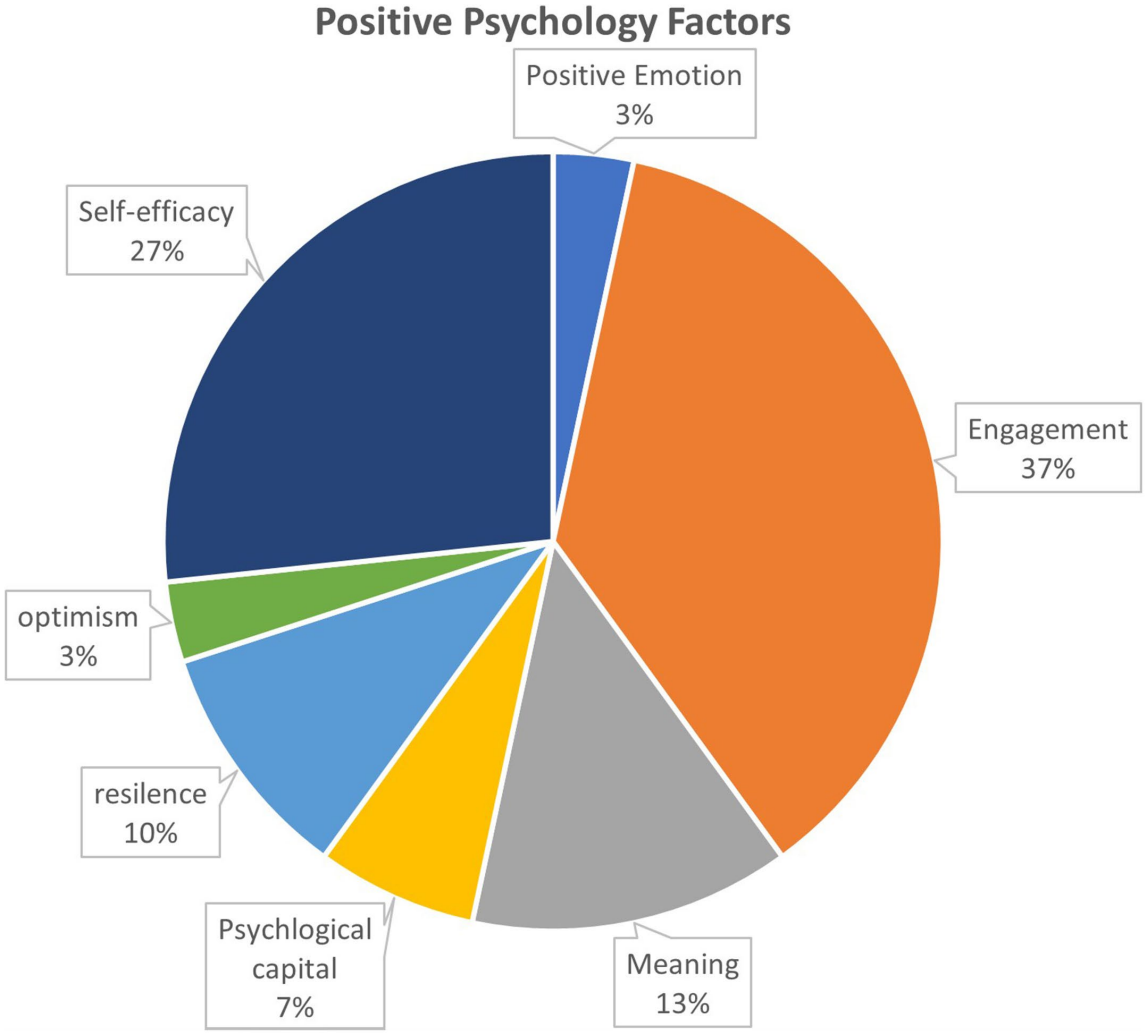
Positive psychology factors discussed in the papers.

Both psychological capital and PERMA are positive psychological resources, and they are conceptualized as being multidimensional and including various psychological aspects (Martínez et al., 2019). In this study, “hope, resilience, self-efficacy and optimism” are the four dimensions of psychological capital, and “Positive emotions, engagement, relationships, meaning and accomplishment” are the dimensions of PERMA. We identify the positive psychology with 7 factors are being discussed 30 times in the previous research (see Figure 3) in the selected articles, some papers use more than 1 elements to conduct the research. The most common used elements are engagement (*n* = 11), self-efficacy (*n* = 8), meaning (*n* = 4) and resilience (*n* = 3) and psychological capital (*n* = 2). There are another 2 papers each on optimism and positive emotion. Hospitality industry, digital technology-based industry, aerospace, bank and travel agencies, healthcare, railway, IT and military organization are the industries that have been separately addressed in different papers. We take this approach to comprehend adaptive performance in various working contexts more thoroughly, which the majority of the previous studies have approved that positive psychology has the significant effect on employee’s behavior and adaptive performance. This study finds that individuals who possess high engagement, self-efficacy or other facets related to positive psychology can have better adaptive performance and work outcome in their career. Adaptive performance has multiple dimensions, according to Pulakos et al. (2002), it has been specified into 8 dimensions, 26 articles out of the selected 27 records in this review adapt the quantitative methodology (including 1 mixed method analysis), 73.08% (*n* = 19) analyze it with uni- dimensional, while the rest 26.92% (*n* = 7) treat it as multi-dimensions.

## 4 Content analysis

### 4.1 Underpin theories

There are 18 theories or models from the 27 peer-reviewed studies that are either cited or used (Table 5). Not all theories arise from the field of positive psychology, for example, the most frequently used theory is conservation of resources theory, driving mechanisms responsible for a variety of stress-related responses and coping strategies (Liao et al., 2022). The other theories that are frequently employed in positive psychology are the self-determination theory, social exchange theory, job demands-resources theory and self-efficacy theory.

**Table 5:** Theories using in the peer-reviewed articles.

Theory	Publication Number
Conservation of resources theory (COR)	6
Not discussed	6
Self-determination	3
Servant leadership theory	2
Social cognitive theory	2
Social exchange theory	2
Career construction theory	1
Job-Demands Resources theory (JDR)	2
Theory of planned behavior	1
Self-regulation theory	1
Social identity theory	1
The broaden-and-build theory	1
The Minnesota Theory of Work Adjustment	1
The theory of passion	1
Theory of self-efficacy	1
Human Capital Theory	1
Transformative learning theory	1
Theory of personality-situation interaction	1
**Total**	**34**

Conservation of resources theory (COR) takes up 18% of the theories using in the peer-reviewed articles, it elucidates the human psychological motivations to protect, acquire, and utilize resources through the continual alteration of a resource’s internal mechanism, which opens up new possibilities for resource depletion and provides a novel perspective to address and recognize stress-related and psychological issues (Tang et al., 2022). The COR theory promotes the development of psychological capital to serve as a conduit or enrichment of the development of other important resources (Al-zyoud and Mert, 2019). According to COR, Luo et al. (2021) explore the formation mechanism of adaptive performance, and the study demonstrates that the psychological capital has a positive effect on employees’ adaptive performance. Another study conducted by Van den Heuvel et al. (2020), it predicts that work engagement trajectories during change are crucial for successful adaptation building on conservation of resources theory.

Three selected papers in this review use the self-determination theory, account for 9% of the total. According to this theory, various goal-directed behavioral norms that are reflective of psychological states influence motivation, and motivation may be intrinsic or extrinsic (Diener, 2009). Extrinsic motivation seems to be less helpful than intrinsic motivation when it comes to an individual’s optimal functioning like happiness and performance. Hamid explains that when an individual’s intrinsic drive and well-being are encouraged, their inherent needs like competence, autonomy, and relatedness can be addressed based on self-determination theory, and they help people to dig out the meaning of job and have positive effect on their work and adaptive performance (Abdul Hamid, 2022). Additionally, the self-determination theory suggests that although behavioral restrictions are distinct, they are arranged along a single continuum of self-determination (Junça-Silva and Menino, 2022).

The broaden and build theory of positive emotions (Vakola et al., 2021) is the another important theory used theories in positive psychology analysis as well. It states that certain positive emotions can expand a person’s ability to think and act in the present moment (Bhambri, 2022). This broadened perspective leads to the building of personal resources like resilience, optimism, and social connections (Xiang and Yuan, 2021), and increase flexibility to help people approach challenges from different angles and find innovative solutions. Meanwhile empirical studies have demonstrated that positive emotions can assist individuals in managing difficult situations (Sriwidharmanely et al., 2021).

Other theories including career motivation theory (Kossek and Perrigino, 2016), self-regulation theory (Bruch et al., 1989), self-efficacy theory (Şahin and Gürbüz, 2014), they focusing on how people motivate and regulate their own behavior in order to achieve their goals. In addition to these theories, other essential concepts are employed to construct research frameworks are person-environment fit theory, job demand-resource theory, social exchange theory (Elshaer and Saad, 2022), the minnesota theory of work adjustment (Griffin and Hesketh, 2003; Lowmiller, 2022), 6 out of 27 papers are found no specific theory applied in their studies.

### 4.2 Direction of causality between positive psychology and adaptive performance

In workplace adaptive performance investigation, researchers employ several methods based on theoretical underpinnings to describe the impact of positive psychology. Research has examined the correlation between adaptive performance and work-related psychological states and has demonstrated a positive correlation between work-related psychological health and adaptive performance (Rowe et al., 2023). First, Psychological capital is the state of mind that motivates and encourages people to reach their full potential, employees who experience positive emotions are able to expand their cognitive abilities, resulting in more imaginative and exploratory thought and action (Luo et al., 2021). Second, talent who with high engagement are more likely to remain motivated despite a decrease in resources, are willing to go above and beyond their duties to meet the objectives of their organization, and are able to compensate for temporary shortages of resources by drawing from larger resources (Bakker and Oerlemans, 2016; Vakola et al., 2021; Kaltiainen and Hakanen, 2022). Vakola et al. (2021) reveal that individuals who are highly engaged in their work have an increased likelihood of adapting to organizational changes, as opposed to those who are more likely to be ambivalent. Third, individuals who view themselves as highly efficacy tend to put in more effort, which, when done correctly, leads to successful results (Şahin and Gürbüz, 2014; Mujeeb et al., 2021). In contrast, Individuals with a low level of self-efficacy are more likely to give up in challenging circumstances and restrict their participation in similar activities (Bruch et al., 1989). And last but not least, job meaningfulness is based on the notion that individuals experience a positive sense of purpose in their work, individual who perceive work as the primary source of meaning and believe that their work contributes to a greater purpose. People search for meaning in their work based on their experience, such as those who acknowledge their presence, their sense of belonging, their relationships, who they are, and their worth and contribution to the work (Van den Heuvel et al., 2020; Abdul Hamid, 2022; Budhiraja and Rathi, 2022; Junça-Silva and Menino, 2022). Hence, job meaningfulness increases employees` sense of purpose and value, thus enabling them to rise to the challenge and foster adaptive performance.

### 4.3 Positive psychology and adaptive performance measurements

#### 4.3.1 Measurement of positive psychology variables

There are total 7 different positive psychology facets that are mentioned and analyzed in these 27 peer-reviewed articles (Table 6) for 34 times, engagement (*n* = 13), self-efficacy (*n* = 9), positive psychology (*n* = 2). Work engagement is a state of contentment and satisfaction associated with work. It is characterized by three dimensions: enthusiasm, commitment, and absorption (Schaufeli et al., 2002). In the study conducted in Indonesia (Nandini et al., 2022), participants were asked to answer a series of questions using the nine-point Utrecht Work Engagement Scale (UwES-9). Within the selected papers, Van den Heuvel et al. (2020) adapt the Utrecht work engagement scale as well, but they measure with six items of Two items per subscale. Another frequent used facet to measure positive psychology in this review study is self-efficacy, an empirical assessment in France (Joie-La Marle et al., 2023) use the 10-item scale, which is designed to measure adaptation and coping abilities, particularly in relation to unforeseen situations (Luszczynska et al., 2005). The original English version of the scale was translated into French through a back translation process due to difficulties in understanding the existing French version. Participants rated the items of the scale on a scale of 1 (absolutely false) to 4 (absolutely true), with scores ranging from 1 to 4 (Joie-La Marle et al., 2023). However, study conducted by Griffin and Hesketh (2003) adapt the 14-item scale to measure participants’ self-efficacy for adaptive behavior, participants were asked to indicate their level of confidence in achieving each of the behaviors at work, ranging from “1” meaning no confidence to “5” meaning very confidence. Jundt et al. (2015) in their review paper summaries that according to Griffin and Hesketh (2003), role-wide self-efficacy was positively associated with self-reported adaptation frequency in the preceding month. Mujeeb et al. (2021) adapt 7-item scale for measure the self-efficacy and confirm that adaptive performance and task performance are not directly impacted by servant leadership, rather self-efficacy has a beneficial effect and acts as a mediator in understanding their relationship. However, according to Pulakos et al. (2002) and Griffin and Hesketh (2003), self-effectiveness for each of the eight dimensions was positively correlated with supervisor ratings of total adaptive performance, but did not demonstrate incremental validity over cognitive capacity and personality (Jundt et al., 2015).

**Table 6:** Positive psychology facets mentioned in the papers.

Variables	Facet	QTY	Sub-total
Independent variable	Psychological Capital	2	17
	Engagement	6	
	Self-Efficacy	5	
	Meaning	2	
	Resilience	2	
Moderator	Meaning	1	2
	Engagement	1	
Mediator	Engagement	6	15
	Meaning	2	
	Resilience	1	
	Optimism	1	
	Self-Efficacy	4	
	Positive emotion	1	
**Total**		**34**	

#### 4.3.2 Measurement of adaptive performance variables

Pulakos et al. (2002) studied 1,000 significant occurrences from 25 job classifications in the U.S. Army and illustrated the worldwide items of adaptive work performance, the scales with 8 dimensions. In the review of the peer 27 articles in this research, Luo et al. (2021) in their 2 articles adapt the measurement items from previous studies with preliminary questionnaire, consisting of 56 statements (ranging from 1 to 5) and participant demos (with 1 indicating strongly disagreement and 5 indicating strongly agree). The scale was translated using the Translation/Back-translation method (McGorry, 2000). The translation was done in English, which was then translated into Chinese with the help of independent bilingual experts, and then re-translated into English to guarantee the quality of the translation. To assess the readiness of the preliminary instrument for use in the present study, four hotel human resources directors and over 50 frontline hotel employees were pretested (Luo et al., 2022). Two behavioral constructs are utilized by another researches (Van den Heuvel et al., 2020) to measure adaptive performance, which are based on adaptive work role performance and extra-role performance. Korean researchers (Park et al., 2020) adapt the shorter version proposed by Charbonnier-Voirin et al., the original scale consists of 19 items, each of which measures five adaptive performance domains, however they select three items from each of the 19 subscales, resulting in a total of 15 items and the reliability of the scale (Cronbach’s α) result to be 0.886. Turkey scholars use originally 8 dimensions scale to measure the adaptive performance (Şahin and Gürbüz, 2014) and two thirds of the articles in this study measure adaptive performance as uni-dimensional (Bruch et al., 1989; Pradhan et al., 2017; Murali and Aggarwal, 2020; Abdullahi et al., 2021; Elshaer and Saad, 2022).

## 5 Conclusion

### 5.1 Overall outcomes

In this study, we provide an SLR to evaluate and synthesize the investigation stream of adaptive performance in a workplace context. Descriptive and substantive results are presented in our bibliography analysis and content analysis, respectively. The papers come from the Web of Science and Scopus databases, the restriction is workplace and without other preference to maximize the search result. We screen the papers and examine them throughout the history of this research.

As a result, 27 papers published from 1989 to 2024 are selected to carry out the systematic literature review to find the most appropriate papers to address our research questions. This study draws upon existing research findings regarding the relationship between positive psychology and employee work adaptive performance. Researchers have looked at conservation of resources theory, self-determination theory, person-environment fit theory and other theories explain the relationship between positive psychology facets and adaptive performance. In addition, this study analyzes the causality relationship of the constructs and reveal the underlying logic why positive psychology of the individual can impact their adaptive performance and work outcome. Measurements of variables are collected and compared in different context. The study finds that there is a significant relationship between positive psychology and employee’s adaptive performance, specifically antecedent positive emotion, engagement, meaning, psychological capital, resilience, optimism and self-efficacy improve the employee’s adaptive performance, empirical data from the banking, IT, hotel, and food and beverage sectors, among others.

### 5.2 The implications for management practice

Individual positive psychology can play an important role for employees to effectively adapting to the changeable working environment, this is the practical implication of this study. Employees who are engaged, or optimistic and self-efficacy are gregarious, determined, and committed, which may offer them the positive energy they need to adapt to change (Van den Heuvel et al., 2020). Understanding that positive psychology is a critical and essential approach, recognizing that work engagement and self-efficacy as a prolonged and continuous process, managers must grasp the pivotal motivational role in fostering positive psychological states and subsequently influencing performance outcomes. It is imperative for employers to proactively furnish employees with training, career opportunities, and rewards, fostering a sense of obligation that prompts elevated levels of adaptive performance (Isah Leontes and Hoole, 2024).

In conclusion, the connection between positive psychology and adaptive performance is effective and multifaceted. The majority of the existing research indicates that the positive psychology elements have a beneficial effect on the employee adaptive performance, however, further empirical research is necessary to determine the extent to which the single or multiple settings can affect the individual adaptive performance. It is critical to consider each of these elements, and cross-disciplinary research is essential in order to further understand the relationship between positive psychology and adaptive performance.

## 6 Limitations and future research

Despite the increasing prevalence of positive psychology and research on employee performance, there is still a lack of research on some aspects of positive psychology, such as “relationships,” “hope” and “accomplishment.” In addition, further empirical studies may be necessary to develop more reliable scales for certain components of the construct, such as self-efficacy, optimism, hope and resilience.

The selection of the research terms and the scope of the research is a limitation of a systematic literature review approach. In this review, only psychological capital, PERMA, and their subsidiary characteristics that were based on prior research are searched for. Peer-reviewed publications in academic journals published in English only are included in this comprehensive literature assessment. This might have limited the availability of pertinent material published in other languages or sources.

In light of the limitations and conclusions of this review, the following areas of future research are proposed in this study. Additional positive psychology ideas should be covered in further reviews, such as well-being, happiness, wellness and peace of mind. In this study, we consider adaptive performance as the dependent variable, researcher may examine if adaptive performance has the opposite impact on positive psychology in subsequent studies. Negative emotions also can be employed as an inverse term and investigate the topic from different perspectives. Academic journals published in languages other than English and a wider range of sources may also be included in the inclusion criteria. During future research, it is possible to conduct a search for relevant articles and examine the documents for which the full text cannot be obtained at this time. And investigations are encouraged to conduct in different contexts, such as different countries and areas, diverse cultures and various industries.

## Author contributions

GT: Writing – original draft, Data curation. RA: Writing – review & editing, Supervision, Formal analysis. SO: Writing – review & editing, Supervision, Methodology.
